# ﻿Waterfalls as a reservoir for caddisfly larvae (Insecta, Trichoptera): exploring a poorly known habitat

**DOI:** 10.3897/zookeys.1263.148087

**Published:** 2025-12-10

**Authors:** Darha Solano-Ulate, Monika Springer

**Affiliations:** 1 Escuela de Biología, University of Costa Rica, San José, Costa Rica University of Costa Rica San José Costa Rica; 2 Museo de Zoología, Centro de Investigación en Biodiversidad y Ecología Tropical (CIBET), University of Costa Rica, San José, Costa Rica University of Costa Rica San José Costa Rica; 3 Centro de Investigación en Ciencias del Mar y Limnología (CIMAR), University of Costa Rica, San José, Costa Rica University of Costa Rica San José Costa Rica

**Keywords:** Aquatic insects, biodiversity, Central America, freshwater habitats, Neotropics

## Abstract

Waterfalls have not been thoroughly studied as a habitat for freshwater macroinvertebrates, although they appear to be an exclusive environment for taxa with traits suited to these unique physical habitat conditions. To better understand the role of waterfalls as aquatic habitats in Costa Rica, macroinvertebrates were collected within the flow and spray zones of 38 waterfalls across the country, spanning an altitudinal range of 55 to 2,660 m above sea level, either by climbing up from the base or using rappel techniques from above. Additionally, in 11 of the waterfalls, corresponding river samples were taken to compare the associated assemblages. Caddisfly larvae were found at all waterfalls sampled, with a total of 10,642 individuals collected from 10 families and 24 identified genera. The family Hydroptilidae, with 12 genera, accounted for half of the individuals collected and was present in 37 of the 38 waterfalls. *Metrichia* (Hydroptilidae) and *Calosopsyche* (Hydropsychidae) were the most abundant genera, in terms of the highest number of individuals, and were most frequently collected, indicating a strong preference for this habitat. Larvae of *Atanatolica* (Leptoceridae) and Xiphocentronidae were quite common in spray zones. In contrast, *Wormaldia* (Philopotamidae), *Contulma* (Anomalopsychidae), *Cerasmatrichia*, and *Alisotrichia* (Hydroptilidae), although uncommon in rivers throughout the country, were also found abundantly in waterfalls, particularly the latter, which were especially abundant in intermittent and karstic waterfalls. Assemblages of Trichoptera in waterfalls were generally similar, with some differences associated with specific site characteristics, such as elevation or rock composition, and chemical factors like conductivity. This research constitutes the first systematic study of caddisfly larvae associated with waterfalls in the Neotropics. The results provide an important baseline for identifying new collection sites of adult caddisflies and for generating associations and descriptions of their larval stages, which may be unknown due to the understudied nature of this habitat.

## ﻿Introduction

Waterfalls are a unique habitat in freshwater ecosystems, characterized by physical characteristics distinct from those of other river habitats, as they lack a water column. Newson and Newson (2000) hydrologically classified this environment based on the flow type as a waterfall, describing it as having torrential water flow in the form of a free fall over the bedrock, at heights greater than one meter and usually covering the entire width of the channel. Additionally, waterfalls provide other microhabitats in hygropetric zones, such as laminar flow over different rock ‘roughnesses’ and spray zones, which usually have associated communities of bryophytes and algae (Zilihona and Nummelin 2001; Korsu 2004; Rackemann et al. 2012; Clayton and Pearson 2016). Although some of their implications for the ecology of certain organisms are known, they are little understood as a habitat in themselves (Rackemann et al. 2012; Baker et al. 2017).

The diversity of aquatic macroinvertebrates associated with waterfalls has been little studied because they represent a habitat challenging to access and sample (Rackemann et al. 2012). Most research on macroinvertebrates in waterfalls has been conducted in the tropics of the Eastern Hemisphere. Some studies have recorded organisms associated with these environments (Yule 1996; Lee et al. 1993; Lee et al. 1998; Lee and Yang 2002; Sites and Vitheepradit 2007; Sites et al. 2011; Wijayanti et al. 2015), while others have evaluated ecological aspects, determining that waterfalls have assemblages distinct from those of the river itself (Palmer et al. 1991; Rackemann et al. 2012; Baker et al. 2016; Clayton and Pearson 2016; Baker et al. 2017). In the Neotropics, studies related to waterfalls have been scarce and mostly limited to specific records of organisms, although there is some knowledge about the preference of certain species for this habitat, specifically for Odonata (Calvert 1915), Hemiptera (Sites et al. 2013; Herrera 2016; Herrera et al. 2020), and the mollusk family Planorbidae (Vogler et al. 2019).

Among the macroinvertebrates that have been recorded associated with waterfalls are the caddisflies (order Trichoptera) (e.g., Palmer et al. 1991; Yule 1996; Rackemann et al. 2012), whose larvae are especially suited to colonize these environments due to their ability to produce silk, which allows them to build shelters and attach themselves in the current (Mackay and Wiggins 1979; Wiggins 2004). The order Trichoptera is highly diverse in the Neotropics, occupying many niches in all freshwater ecosystems (Pes et al. 2018). Although adult diversity is relatively well understood in certain neotropical countries (e.g., Armitage et al. 2024), larval stages remain understudied and are unknown for most species, as well as several genera (Springer 2010; Pes et al. 2018).

Despite their important scenic and recreational value for people, and even economic value as tourist attractions (Hudson 2013; Clayton and Pearson 2016; Ortega-Becerril et al. 2019), waterfalls, like freshwater environments in general, are threatened by multiple anthropogenic activities, as well as by climate change (Dudgeon et al. 2006; Rackemann et al. 2012; Sayer et al. 2025). Therefore, it is important to guarantee their conservation and to understand their contribution to regional biodiversity. This work aims to contribute to the knowledge of the caddisfly fauna inhabiting waterfalls in a neotropical country, highlighting the importance of these understudied environments as habitats for the local freshwater fauna.

## ﻿Materials and methods

### ﻿Study area

This study was conducted in Costa Rica, located on the southern Central American isthmus and part of the Neotropical region. Due to its position between the Pacific and Atlantic Oceans and its central mountain system, the country exhibits considerable topographic and climatic diversity within a relatively small area of 51,100 km^2^. These conditions result in high environmental heterogeneity and varying rainfall patterns, ranging from 600 mm in the Northern Pacific lowlands to more than 7,000 mm of annual average rainfall along the Caribbean slope and the southern Pacific area (Avalos 2019). Its hydrographic system is divided into 34 basins that drain through three watersheds: The San Juan River in the North, the Caribbean Sea, and the Pacific Ocean. Rivers and streams are abundant throughout the country, and due to the diverse topography, hundreds of waterfalls can be found at all elevations.

### ﻿Data collection

**Figure 1.**
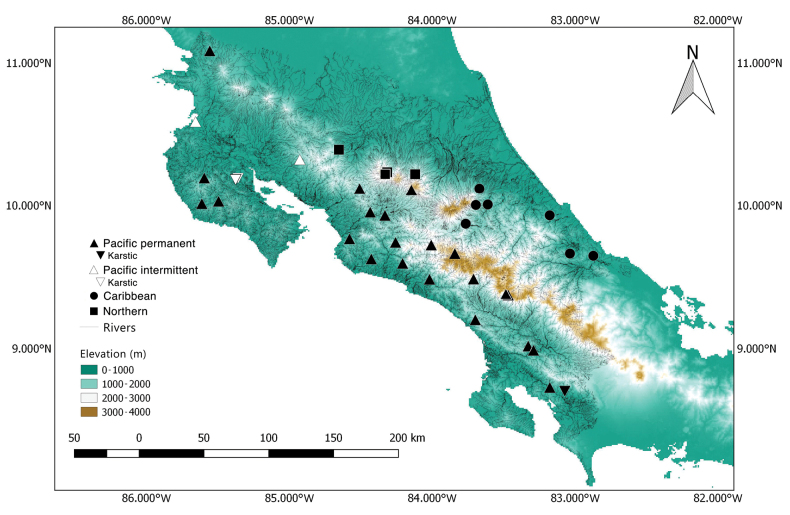
Map of study sites. Geographical location of sampling sites in Costa Rica, according to their slope distribution. Refer to Table 1 for site details.

**Figure 2.**
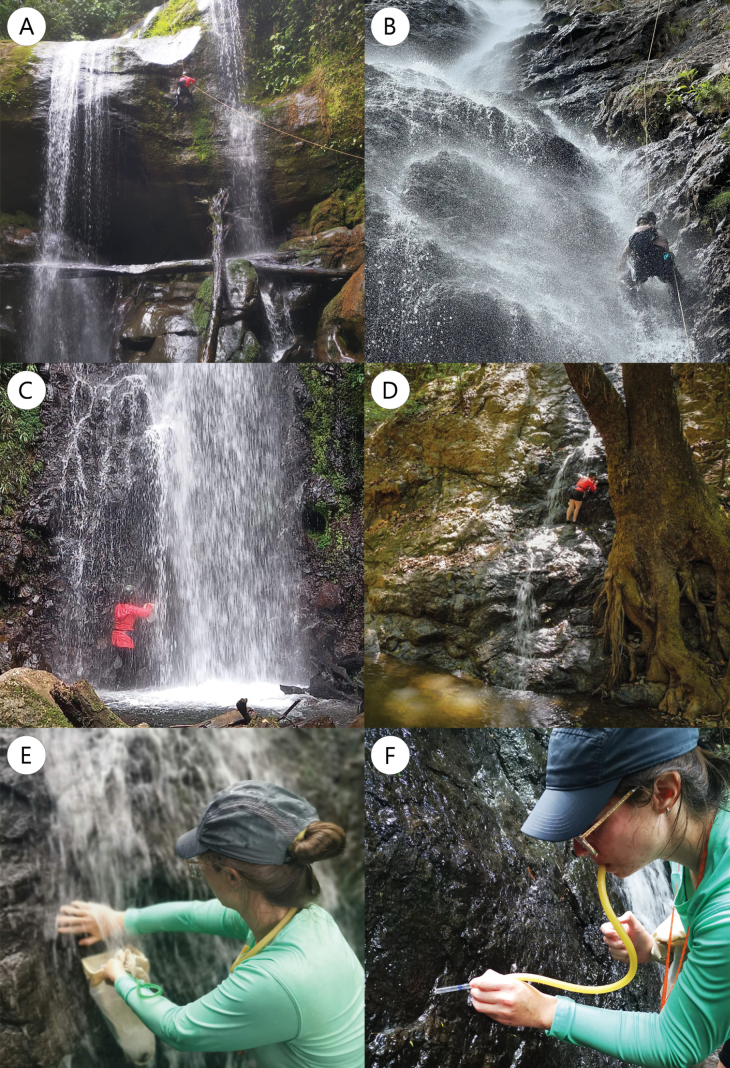
Collecting techniques and microhabitat sampling methods. Techniques used for collecting waterfall macroinvertebrates: **A.** Guided rappel; **B.** Rappel; **C, D.** Sampling from the base; **E.** Flow collecting with a hand net; **F.** Direct collecting with an entomological aspirator and forceps in spray areas.

A total of 38 waterfalls, with estimated heights ranging from 4 to 140 m, were sampled throughout the country, located between 55 and 2,660 m a.s.l. (Fig. 1; Table 1). Waterfalls were selected based on accessibility, their location mainly within well-preserved forested areas, and their representation of all three drainage areas (Northern, Pacific, and Caribbean slope) across the country’s altitudinal range. Thirty-four waterfalls have permanent flow, while four in the North Pacific dry forest region are intermittent (B2, B6, B7, and B8), becoming completely dry for several months, generally between January and late April. Two of the four intermittent waterfalls (B6 and B7) are karstic and share this characteristic with R27, one of the permanent waterfalls in the Southern Pacific lowland. Sampling took place between January 2022 and April 2023, and two collecting techniques were used: i) rappelling from the top of the waterfalls using appropriate equipment (Fig. 2A, B), and ii) sampling directly from the base, climbing the waterfall wall when possible (Fig. 2C, D). All waterfalls were sampled once; eleven were sampled by rappelling, and 27 were sampled from the base. In all cases, sampling took place at least 1 m above the base to avoid contact with the riverbed microhabitats.

**Table 1.** Site information and physical and chemical data. Site codes of the 38 waterfalls sampled identified by sampling technique, intermittency, and karstic rock composition (B = Base, R = Rappel, i = intermittent, k = karstic), geographical coordinates, slope (P = Pacific, C = Caribbean, N = Northern), elevation and physical and chemical parameters measured in situ (h estim. = height estimated, T = temperature, DO = dissolved oxygen, Cond. = conductivity, ND = not determined).

Site code	Coordinates (DD)	Slope	Elevation (m a.s.l.)	h estim. (m)	T (°C)	pH	DO (mg/L)	Cond. (µS/cm)
B1	11.05756; ⎯85.58302	P	197	4	24.8	7.49	6.5	187.9
B2 i	10.56262; ⎯85.67559	P	55	6	26.9	8.34	7.1	374
B3	10.16276; ⎯85.60492	P	263	15	24.1	6.66	6.0	156.0
B4	9.99378; ⎯85.50336	P	234	10	ND	ND	ND	ND
B5	9.97609; ⎯85.62225	P	60	8	24.3	7.28	5.9	223
B6 ik	10.18462; ⎯85.36526	P	125	6	25.7	8.17	7.9	432
B7 ik	10.17434; ⎯85.37022	P	148	6	25.5	8.13	7.6	530
B8 i	10.29407; ⎯84.93556	P	360	10	24.7	7.94	6.9	165.3
R9	10.09342; ⎯84.51335	P	900	60	20.4	ND	8.3	98.5
R10	9.92698; ⎯84.44930	P	345	80	ND	ND	ND	ND
B11	10.07774; ⎯84.15257	P	1,445	5	17.7	7.04	8.3	73.8
R12	9.72049; ⎯84.26418	P	810	120	22.0	7.56	6.1	145.9
R13	9.90716; ⎯84.33932	P	430	25	ND	ND	ND	ND
R14	9.74341; ⎯84.58826	P	350	140	ND	ND	ND	ND
B15	9.60658; ⎯84.43174	P	85	10	26.0	8	6.9	174.4
B16	9.57920; ⎯84.21212	P	188	4	25.0	8	8.1	181.2
B17	9.47057; ⎯84.02183	P	450	5	25.5	7.5	7.8	114.8
B18	9.64511; ⎯83.85177	P	2,660	8	12.3	7.16	7.6	40.3
B19	9.69975; ⎯84.01320	P	1,900	10	16.4	4.8	8.2	55.1
B20	9.46822; ⎯83.72358	P	1,438	10	17.4	7.5	8.5	ND
B21	9.18186; ⎯83.70674	P	150	4	27.0	8	7.7	ND
R22	9.36995; ⎯83.48675	P	1,695	7	15.8	8.03	9.4	118.3
R23	9.37193; ⎯83.48308	P	1,820	50	16.0	8.07	8.8	118.8
R24	8.99363; ⎯83.32425	P	535	50	26.3	8	7.6	77.6
B25	8.97481; ⎯83.29524	P	96	10	25.8	8	6.7	407
B26	8.70710; ⎯83.17583	P	84	5	26.5	8	8.2	122.9
R27 k	8.71162; ⎯83.06985	P	190	25	ND	ND	ND	ND
R28	9.99041; ⎯83.70276	C	1,408	45	18.2	7.41	7.1	70.2
B29	9.86219; ⎯83.77659	C	970	7	ND	ND	ND	ND
B30	10.00475; ⎯83.61945	C	412	7	24.5	7.93	8.4	258
B31	10.11916; ⎯83.68175	C	627	7	18.5	7.95	8.4	45.6
B32	9.92423; ⎯83.18555	C	245	10	26.1	8.27	7.2	374
B33	9.65752; ⎯83.03632	C	215	60	25.7	8.41	7.7	110.9
R34	9.64289; ⎯82.87025	C	115	40	25.0	8.35	7.8	254
B35	10.20921; ⎯84.12853	N	1,610	5	16.3	8.25	8.0	41.3
B36	10.21382; ⎯84.32641	N	1,840	30	15.7	7.59	8.3	47.8
B37	10.20888; ⎯84.32899	N	1,770	7	16.0	7.45	9.0	48.5
B38	10.37733; ⎯84.66341	N	777	10	21.3	7.5	8.1	153

For both collecting techniques, in each waterfall, the same microhabitats were qualitatively sampled, including both the direct flow and the spray areas. A hand net with a 250-µm mesh size was used to sample microhabitats with turbulent and/or laminar flow, while spray areas were sampled directly using forceps and an aspirator (Fig. 2E, F). Four physical and chemical parameters were measured *in situ* in 32 sites: temperature, pH, dissolved oxygen, and direct conductivity, using a PCE-PHD 1 multiparameter and Macherey-Nagel pH test strips (Table 1). Waterfall heights were estimated using the rope length in rappel sampling, and photographs with the collector present as scale for base-sampled waterfalls.

Additionally, 11 waterfalls were selected of the 38 to compare caddisfly diversity and abundance between this habitat and the river caddisfly assemblages to identify any association or preference for this particular habitat. In these 11 rivers, macroinvertebrates were collected qualitatively either upstream or downstream, depending on access to the site, mainly in riffles using a D-net with a 500 µm mesh size. All samples were preserved in the field in 95% ethanol and collected under R-SINAC-SE-DT-PI-003-2021 and R-SINAC-SE-DT-PI-007-2022 permits.

Samples with collected organisms were sorted in the laboratory using a stereomicroscope and identified to the lowest taxonomic level possible (mainly genus), using specialized literature and local identification keys for Trichoptera larvae (Springer 2010; Pes et al. 2018). All specimens were deposited in the aquatic entomology collection of the Zoology Museum at the University of Costa Rica (MZUCR).

### ﻿Data analyses

Since waterfall collecting was qualitative, Trichoptera abundance data were converted to relative abundances for all the statistical analyses. To compare Trichoptera diversity between waterfall and river habitats, relative abundances were analyzed using a Wilcoxon paired test for each taxon to determine the associations of taxa with each habitat. To examine the relationship between all the waterfall Trichoptera assemblages, a cluster analysis was performed using the Bray-Curtis similarity index. To explore the patterns of caddisfly diversity among sites, an unconstrained non-metric multidimensional scaling (NMDS) was conducted using the Bray-Curtis dissimilarity index. The environmental variables measured at the 32 sites (temperature, pH, dissolved oxygen, and conductivity) plus elevation were then correlated with their corresponding axis values using multiple linear regression. Significant environmental variables were incorporated as covariates in a second NMDS to improve the environmental explanation of the ordination pattern. Environmental data were previously transformed using Z scores. Analyses were performed using the software Past 5.2.1 (Hammer et al. 2001).

## ﻿Results

Among the ten insect orders found in the 38 waterfalls, Trichoptera was the second most abundant group, after Diptera. It represented 33% of all collected macroinvertebrates, with 10,608 individuals from ten families and 24 identified genera (Table 2). Caddisfly larvae were present in all 38 waterfalls, with abundances of 9–948 individuals collected per site, while taxa richness ranged from 1 to 9 families, and 1–14 genera (Suppl. material 1).

**Table 2.** Taxonomic list of caddisflies in samples. Count of individuals (abundance) of caddisfly larvae collected per genus in the total waterfalls, waterfall subsample, their corresponding rivers samples, and quantity of waterfalls where each taxon was found (Freq.).

Family	Genus	Total Abundance			Freq.
		Waterfalls (*n* = 38)	Waterfalls (*n* = 11)	River (*n* = 11)	
Anomalopsychidae	* Contulma *	92	48	3	8
Calamoceratidae	* Phylloicus *	–	–	45	–
Ecnomidae	* Austrotinodes *	–	–	20	–
Glossosomatidae	Gen. Undet.	2	–	440	2
Helicopsychidae	* Helicopsyche *	129	81	57	13
Hydrobiosidae	* Atopsyche *	69	19	65	11
Hydropsychidae	* Calosopsyche *	2,411	916	108	30
	* Leptonema *	47	7	182	12
	* Smicridea *	46	3	477	10
	Gen. Undet.	14	12	–	2
Hydroptilidae	* Alisotrichia *	2,214	1	1	16
	* Anchitrichia *	1	–	7	1
	* Byrsopteryx *	233	81	3	16
	* Cerasmatrichia *	62	23	4	15
	* Hydroptila *	2	1	24	2
	* Leucotrichia *	112	96	39	19
	* Mayatrichia *	3	3	4	1
	* Metrichia *	2,106	509	125	33
	* Neotrichia *	16	10	322	8
	* Ochrotrichia *	66	64	1	5
	* Oxyethira *	–	–	8	–
	* Rhyacopsyche *	133	39	22	18
	* Zumatrichia *	74	74	3	10
	Gen. Undet.	531	345	–	11
Lepidostomatidae	* Lepidostoma *	57	2	198	2
Leptoceridae	* Atanatolica *	629	321	14	15
	* Nectopsyche *	3	1	279	3
	* Oecetis *	–	–	38	–
	* Triplectides *	–	–	11	–
Odontoceridae	* Marilia *	–	–	36	–
Philopotamidae	Chimarra (Chimarra)	4	4	181	1
	Chimarra (Curgia)	1,284	167	–	17
	* Wormaldia *	127	13	–	8
	Gen. Undet.	–	–	2	–
Polycentropodidae	* Polycentropus *	–	–	70	–
	* Polyplectropus *	–	–	3	–
Xiphocentronidae	Gen. Undet.	141	36	9	29

Hydroptilidae was the most frequently occurring caddisfly family in waterfalls, present in 37 of the 38 waterfalls sampled, with the exception of site B19 (Supp. material 1). Also, Hydroptilidae had the highest abundance with > 5,000 individuals, accounting for almost half of all caddisflies collected, as well as the highest taxonomic diversity with 12 identified genera (Table 2). *Metrichia* was the most frequently encountered genus in 33 waterfalls, with a little > 2,100 individuals. Its abundance was higher in waterfalls located at low and middle elevations. Also highly abundant was *Alisotrichia*, with > 2,150 individuals, although it was less widespread, as this hydroptilid was found in only 16 waterfalls, primarily located in lowland areas. The second most abundant and frequent family was Hydropsychidae, comprising > 2,500 individuals. *Calosopsyche* was the most frequently collected genus within this family, present at 30 waterfalls with 2,411 individuals (Table 2). Also abundant was Chimarra (Curgia) (Philopotamidae), with > 1,200 individuals collected from 17 waterfalls. However, it was only widespread on the Pacific slope, while a few organisms of Chimarra (Chimarra) were collected at a single site on the Caribbean slope (Suppl. material 1).

Family Xiphocentronidae and genus *Atanatolica* (Leptoceridae) were commonly found in the spray areas of waterfalls, while the genera *Atopsyche* (Hydrobiosidae) and *Contulma* (Anomalopsychidae) (Fig. 5F) were occasionally collected in a wide altitudinal range, and their highest abundances were obtained in intermediate to high elevations. On the other hand, the family Glossosomatidae and six additional genera were collected in fewer than three waterfalls, with *Lepidostoma* (Lepidostomatidae) and *Hydroptila* (Hydroptilidae) collected only in two sites, and *Anchitrichia* and *Mayatrichia* (Hydroptilidae) found in only one waterfall and with < 5 individuals each (Table 2; Suppl. material 1).

**Figure 3.**
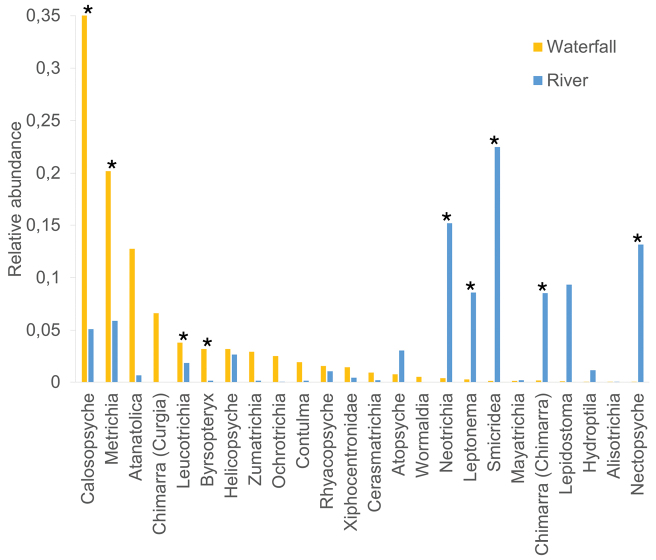
Relative abundances of Trichoptera in waterfalls versus river habitats. Comparison of the relative abundance of caddisfly larvae shared between 11 waterfalls and their corresponding rivers. Taxa abundance significantly different (p < 0.05) between habitats, as determined by the Wilcoxon test, is marked with an asterisk (*; refer to Suppl. material 1 for the Wilcoxon test results).

At the 11 sites where both waterfall and river caddisfly assemblages were sampled, a higher taxonomic richness was observed in the rivers, comprising 14 families and 29 genera, which corresponded to 2,644 individuals (Table 2). Twenty-three taxa (21 genera and the families Glossosomatidae and Xiphocentronidae) were shared between the total waterfall samples and the river samples. The families Calamoceratidae, Ecnomidae, Odontoceridae, Polycentropodidae, and genus *Oxyethira* (Hydroptilidae) were only found in rivers, while the Philopotamidae genus *Wormaldia* and subgenus Chimarra (Curgia) were exclusively collected in waterfalls (Fig. 5B, C; Table 2), inhabiting the same type of shelters and habitus. Also, some of the less abundant taxa in waterfalls (i.e., Glossosomatidae; Chimarra (Chimarra); *Smicridea*) were very abundant in rivers, while others, like *Anchitrichia* and *Mayatrichia*, were rarely found in either habitat (Fig. 3; Table 2). Based on the Wilcoxon paired test, the genera *Calosopsyche*, *Metrichia*, *Leucotrichia* and *Byrsopteryx* were associated with waterfalls habitat, while *Neotrichia*, *Leptonema*, *Smicridea*, *Nectopsyche*, and subgenus Chimarra (Chimarra) were associated to river habitat (Fig. 3; Suppl. material 1). *Helicopsyche* abundances were similar between waterfalls and rivers; however, the case shapes observed in the waterfall specimens were very different, and they were found in spray areas (Figs 3, 5G).

**Figure 4.**
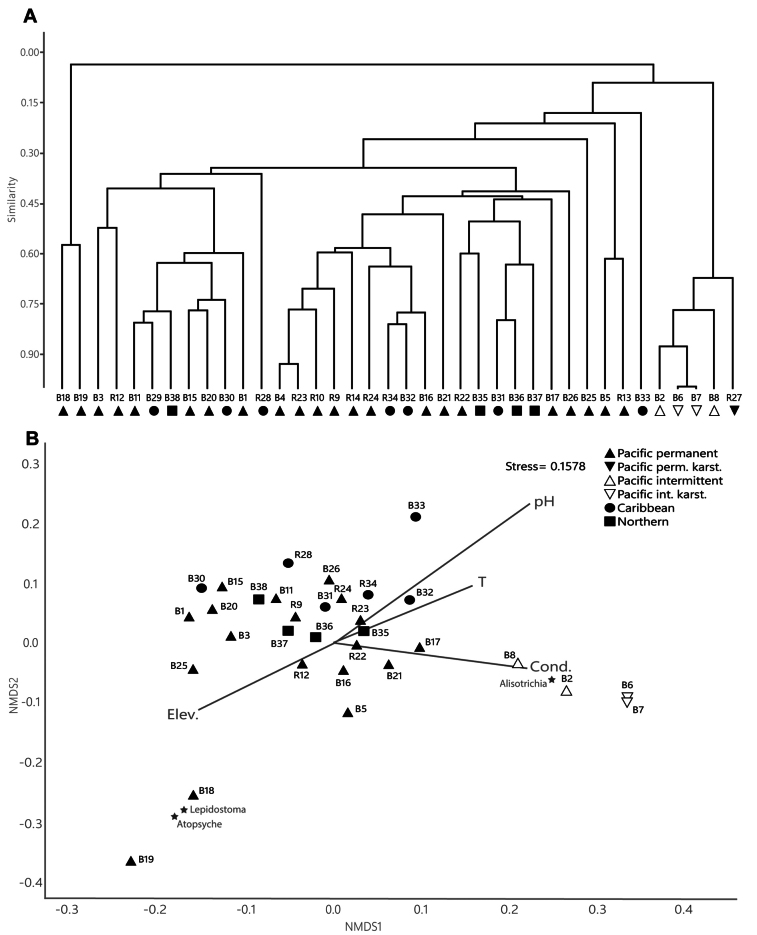
Cluster analysis and NMDS. **A.** Cluster analysis based on the Bray-Curtis similarity index of Trichoptera assemblages in the 38 waterfalls samples; **B.**NMDS ordination plot in two dimensions of 32 sites with significantly fitted vectors of environmental variables (elevation, temperature, pH, and conductivity). The three most differentiated taxa are also shown in the ordination plot.

The cluster analysis revealed three main groups of waterfall Trichoptera assemblages (Fig. 4A). The first group consisted of sites B18 and B19, which were located at the highest elevations and shared the presence of the genera *Atopsyche* and *Lepidostoma* (Table 1; Suppl. material 1). The second group was formed by all the North Pacific intermittent and karstic sites (B2, B6, B7, and B8) and the karstic South Pacific site (R27), sharing approximately 65% assemblage similarity and the highest abundances of the genus *Alisotrichia* (Fig. 4A; Suppl. material 1). A third group was formed by all remaining sites, sharing different levels of similarity and slope locations (Fig. 4A).

**Figure 5.**
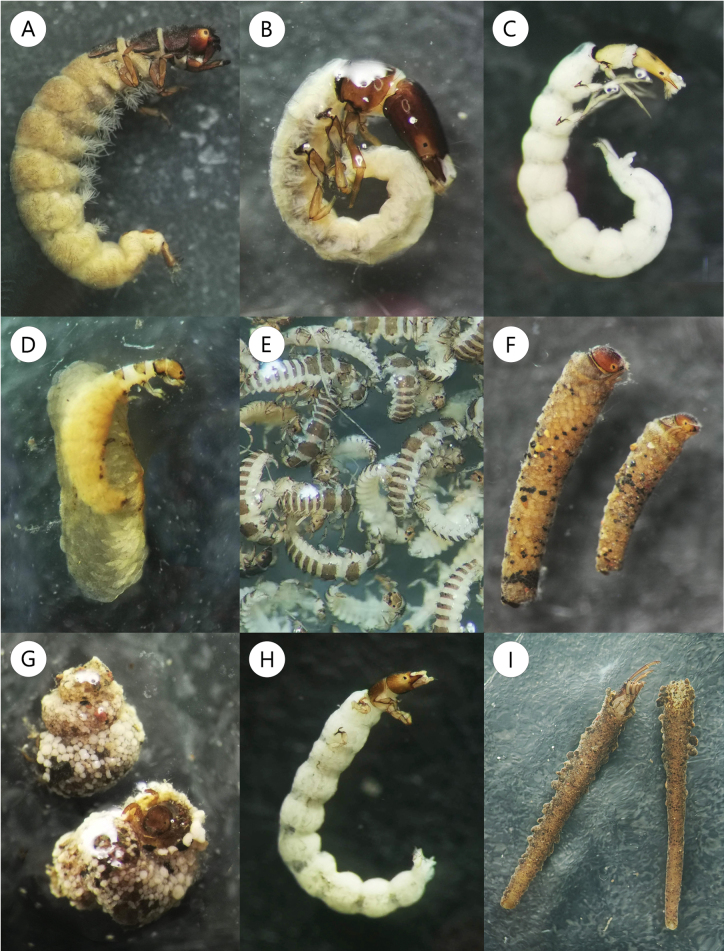
Examples of Trichoptera larvae collected from waterfalls in Costa Rica. **A.***Calosopsyche* (Hydropsychidae); **B.**Chimarra (Curgia), and **C.***Wormaldia* (Philopotamidae); **D.***Metrichia*, and **E.***Alisotrichia* (Hydroptilidae); **F.***Contulma* (Anomalopsychidae); **G.***Helicopsyche* (Helicopsychidae); **H.**Xiphocentronidae; **I.***Atanatolica* (Leptoceridae).

The physical and chemical measurements varied among sites, differing according to location. Temperatures varied between 12.3 °C for the higher elevation sites and 27 °C for the lowland waterfalls. Conductivity ranged from 40.3 to 530 µS/cm, while pH values varied from 6.66 to 8.41, and one site exhibited a low pH of 4.8 (Table 1). Four of the five environmental variables analyzed with the multiple linear regression were significantly correlated with the sites ordination in the first NMDS conducted, elevation (R2 = 0.12, p = 0.04), temperature (R2 = 0.13, p = 0.04), pH (R2 = 0.23, p < 0.001) and conductivity (R2 = 0.28, p < 0.001). Sites ordination in the NMDS, including these covariates, reflected similar patterns as observed in the cluster analysis (Fig. 4B), differentiating the first group associated with high altitude and low pH values, the second group with high conductivity values, and showing no strong patterns in the ordination of the third sites group. The three genera mentioned earlier were also highly differentiated in the NMDS plot ordination between the first and second groups, as well as the third.

## ﻿Discussion

The overall composition of macroinvertebrate assemblages from Costa Rican waterfalls showed some similarities with those observed in studies of waterfalls in other regions, where the orders Diptera and Trichoptera were the most dominant taxa (Palmer et al. 1991; Yule 1996; Baker et al. 2016; Clayton and Pearson 2016; Dávila-Recinos et al. 2019). Dominance of families Hydroptilidae and Hydropsychidae observed in waterfalls in Costa Rica was similar to that observed in studies of waterfalls in other regions (Palmer et al. 1991; Yule 1996; Rackemann et al. 2012; Baker et al. 2016; Clayton and Pearson 2016; Dávila-Recinos et al. 2019). The groups identified in this study primarily correspond to highly rheophilic organisms, adapted to life in the current (Rackemann et al. 2012; Clayton and Pearson 2016), as is the case with caddisflies, due to their use of silk (Wiggins 2004).

The diversity of caddisflies collected at waterfalls in the present study included ten of the 15 families recorded from Costa Rica (Springer 2010). Hydroptilidae presented the highest diversity and abundance, as well as the greatest frequency. Costa Rica has 17 reported genera of Hydroptilidae (Armitage et al. 2024), of which 12 were collected from the sampled waterfalls. *Metrichia*, the most frequent and abundant genus found in this study (Fig. 5D), is one of the most species-rich hydroptilid genera in the Neotropics, and its larvae are associated with fast currents and the presence of filamentous algae (Pes et al. 2018; Desidério et al. 2023). The waterfalls where *Metrichia* was most abundant were located at medium and low elevations, characterized by warm temperatures and high exposure to sunlight. Moreover, some were located in environments with agricultural land use, which favors the proliferation of filamentous algae. Together with the torrential flow of the waterfalls, these conditions appear to create a favorable environment with abundant food availability, thereby facilitating the development of *Metrichia* larvae, as shown by the habitat comparison (Fig. 4). Interestingly, the only site where Hydroptilidae was not collected presented the lowest pH, with 4.8 (Table 1). This suggests that a low pH could be a limiting factor for colonization by hydroptilid larvae, although larvae of this family have been collected from rivers with even lower pH values (unpublished data, based on the National Monitoring Program).

On the other hand, although less frequent, the genus *Alisotrichia* was found mainly in lowland waterfalls and abundantly in all the intermittent and karstic waterfalls (Fig. 5E). In the NMDS, a relationship was observed between the presence of *Alisotrichia* larvae and the increase in conductivity, together with the ordering of the intermittent waterfalls (Fig. 4). Sampling at these sites was conducted during the rainy season, so the measured conductivity values were high (Table 1), possibly due to the high sediment and mineral load carried by the current as a result of runoff and also associated with the karst composition of some of these waterfalls. *Alisotrichia* larvae are free-living and inhabit rocks in fast-flowing rivers with laminar flow, or spray areas around waterfalls (Springer 2010; Alves et al. 2023), where they feed on microalgae scraped from the substrate (Springer 2010). There may be a relationship between the high solute load and the development of microalgae or bacteria on which these caddisflies feed, thereby favoring the presence of large quantities of these organisms, especially in lowland areas. Similar conditions could also favor other hydroptilids, such as *Byrsopteryx and Leucotrichia*, which showed a preference for waterfall habitat (Fig. 3), and *Cerasmatrichia*, *Rhyacopsyche*, and *Zumatrichia*, despite they may not have a habitat preference, which were all found in more than ten waterfalls and collected in higher numbers than in their corresponding rivers (Table 2).

*Neotrichia* was occasionally found in waterfalls, but its abundance was higher in the sampled rivers, and with the habitat comparison, it is possible to confirm that these environments are its preferred habitat (Fig. 3). In contrast, species of *Hydroptila*, *Anchitrichia*, and *Mayatrichia* were found in only one or two waterfalls, and their presence in these waterfalls may represent incidental collections. In the case of *Mayatrichia*, this genus is also not frequently collected in rivers, and could have a very restricted distribution. All hydroptilid genera collected in rivers were also found in waterfalls, except for *Oxyethira*, which was collected only in rivers, as expected, given its habitat, since this caddisfly is more common in slow-flowing rivers or even lentic environments (Springer 2010). It is known that hydroptilid larvae in general have an affinity for living mainly in spray-zone areas and high current velocities in rivers and waterfalls, where they feed on the periphyton and microalgae that they scrape from the substrate (Springer 2010; Pes et al. 2018), so the high diversity of hydroptilids collected in waterfalls was expected.

Larvae of genus *Calosopsyche* (Hydropsychidae) were dominant both in abundance and presence in the studied waterfalls (Fig. 5A). There is not much information on the ecology of this generally rarely collected genus. However, it is known to have a preference for high mountain streams with high oxygenation where it can be locally abundant (Springer 2010). As they were so common in waterfalls, the habitat analysis showed that this is likely their preferred habitat (Fig. 3), as the turbulent flow can meet the requirement for high oxygen concentrations. Interestingly, the genera *Leptonema* and *Smicridea* were found in waterfalls at much lower abundances and frequencies, despite their high abundance in rivers (Table 2) and much higher species richness (Holzenthal and Calor 2017), the habitat comparison corroborate its preference for river habitat (Fig. 3). These hydropsychid larvae feed on organic matter, which they filter through silk webs where they also take refuge (Springer 2010; Pes et al. 2018). The changes in abundance observed between these three genera (Fig. 3) suggest a specialization by habitat type, allowing niche partitioning to avoid competition between organisms for the same resources. Differences in microhabitat distribution and growth patterns have also been observed between hydropsychid species with equivalent niches in temperate areas (Hauer and Stanford 1982).

The family Philopotamidae was represented in waterfalls by two genera, *Chimarra* and *Wormaldia* (Fig. 5B, C). *Chimarra* is widespread and very common in rivers, with a larger number of described species distributed across four subgenera (Flint 1998; Blahnik and Holzenthal 2012; Pes et al. 2018). Of the 18 waterfalls where this genus was collected, the subgenus Chimarra was found only at one site on the Atlantic slope, while the other 17, all located on the Pacific slope, corresponded to the subgenus Curgia, which was confirmed by the emergence of an adult. On the other hand, the genus *Wormaldia* was less abundant and frequent in waterfalls and is generally considered rare (Springer 2010). Larvae are known to inhabit wet areas on rocky surfaces in forested areas, where they build bag-like silk shelters that retain particles of organic matter on which they feed (Springer 2010; Muñoz-Quesada and Holzenthal 2015; Pes et al. 2018). Both genera were observed forming the same type of shelters on waterfall walls, in laminar flow and spray zones, where they were maintained with a constant drip of water. Of the caddisflies found in the waterfalls, Chimarra (Curgia) and *Wormaldia* were not collected in the corresponding rivers, while Chimarra (Chimarra) was abundant in rivers (Fig. 3; Table 2), suggesting that waterfalls are the preferred habitat for both Chimarra (Curgia) and *Wormaldia*.

Xiphocentronidae were also very frequent in waterfalls, especially in spray zones where they were collected in greater abundance (Fig. 5H). These caddisflies are known to be highly adapted to inhabiting spray areas of rivers (Pes et al. 2018) and are widespread and abundant in rivers and streams throughout Costa Rica. Unfortunately, the taxonomic identification of the larvae is challenging, as no diagnostic characteristics have been identified to date that enable specimens to be identified to the genus level (Pes et al. 2018). Another common caddisfly in spray zones was *Atanatolica* (Leptoceridae) (Fig. 5I). From the family Leptoceridae, *Atanatolica*, in particular, is known to inhabit waterfall walls or large boulders, mainly in rivers with minimal environmental alteration, where it feeds by scraping algae from the substrate (Holzenthal 1988; Springer 2010). On the other hand, only a few individuals of the leptocerid *Nectopsyche* were collected at just three sites, and their abundance was higher in the river samples; thus, the habitat comparison revealed their preference for this habitat (Fig. 3; Table 2). Both Xiphocentronidae and *Atanatolica* were more abundant in waterfalls; however, they could also be equally abundant in rivers. Due to their specific microhabitat, they are not typically collected using traditional methods; instead, they require direct collection from the substrate.

Another genus commonly found in the spray areas of lowland waterfalls was *Helicopsyche* (Helicopsychidae). These caddisflies build spiral-shaped shelters from grains of sand and are commonly found in slow to moderately flowing rivers (Springer 2010; Martín and Miranda 2015). The larvae collected in this study from the walls of waterfalls differ significantly in the shape of their shelters, which feature a higher spiral in their cases than the more flattened cases typically found in river specimens (Fig. 5G). There is a previous mention of *Helicopsyche* larvae collected in hygropetric environments in the Caribbean (Botosaneanu, 1996). Still, no further information could be found on the habits of these larvae in waterfalls specifically. Therefore, this could also be a previously undescribed attribute related to a particular species (Botosaneanu 1996).

The genera *Atopsyche* (Hydrobiosidae), *Contulma* (Anomalopsychidae), and *Lepidostoma* (Lepidostomatidae) were found at the highest elevation sites. *Atopsyche* larvae are predatory and prefer river habitats with fast currents where they are frequently encountered (Springer 2010; Pes et al. 2018). Due to these characteristics, some individuals may be present at the waterfalls, taking advantage of the habitat to search for food. At site B18, 40 individuals were counted, representing the main predators, given the low diversity of macroinvertebrates typically encountered, which is likely associated with the high altitude (Jacobsen 2003). In site B19, despite its abundance of only seven individuals, *Atopsyche* also constituted the main predator for the same reason. Both sites were associated in the cluster analysis, as evidenced by the shared caddisfly, and also had the highest elevation among all waterfall samplings (Fig. 4).

In the case of Anomalopsychidae, a family with few species in two genera, of which only *Contulma* is found in Costa Rica (Holzenthal and Calor 2017), larvae have been rarely collected throughout the country (information based on the aquatic entomology collection at MZUCR). *Contulma* species are primarily found in small streams, waterfalls, or seeps at high altitudes (Holzenthal and Flint 1995; Holzenthal and Calor 2017). The waterfalls where the genus was collected with relatively high abundances in this study were located at altitudes above 1400 m a.s.l., with the exception of one at 345 m, where only two individuals were found (Suppl. material 1). Although there was no significant difference between waterfall and river habitats (Fig. 3), *Contulma* may be more closely related to waterfall habitats, as only three individuals were collected in rivers (Table 2). This could also explain its low representativeness in the collection, where samples from waterfalls are rare.

Contrary to *Contulma*, larvae of *Lepidostoma*, which were collected only from waterfalls B18 and B19 at the highest elevations, had a much greater abundance in the river habitat (Table 2). *Lepidostoma* also contributed to the ordination of the sites in the analyses (Fig. 4). This genus has only a few species in Costa Rica, almost all reported from higher altitudes, and the individuals collected at the waterfalls could represent a species specialized for this habitat since it showed distinct morphological characteristics compared with those found in rivers. In contrast, the presence of Glossosomatidae, which was also encountered at only two waterfalls, is considered an incidental collection, as only two early-stage larvae were collected, whereas this family is very abundant and widely distributed in rivers and streams throughout the country (Springer 2010).

Of all the caddisfly families collected in the rivers, Calamoceratidae, Ecnomidae, Odontoceridae, and Polycentropodidae were not found in any of the studied waterfalls. These families are mainly associated with slow-flowing sections, riverbanks, or pools (Springer 2010). In the case of Ecnomidae, it is noteworthy that they were found relatively abundant at the B19 site, even though this family is rarely collected throughout the country (information based on the aquatic Entomology collection at MZUCR). This site has the lowest pH value measured, which also influenced the NMDS sites ordination (Fig. 4B). Little is known about Ecnomidae and its environmental associations (Springer 2010), and a possible relation with this particular factor may be considered for further studies on this family. Of the 15 families of caddisflies in Costa Rica, Limnephilidae was the only one not collected during this study, as the distribution of its only reported species is restricted to the country’s highlands, and it prefers low-flow or lentic environments (Springer and Bermúdez 2018).

Almost all of the Trichoptera found in the waterfalls corresponded to taxa that can also be found in rivers, some more frequently than others. As Palmer et al. (1991) mentioned, all the river biotopes are areas connected to each other along and across their course, so it cannot be expected that each biotope will harbor a completely different diversity from the other, but rather the organisms will be distributed among them with greater or lesser preference. Some caddisflies considered rare in rivers may be very common in waterfalls (e.g., *Contulma*, *Calosopsyche*). They are generally rarely collected, possibly because they have a more restricted distribution, and waterfalls are often not included as sampling habitats in ecological studies of aquatic environments. For some organisms, waterfalls may represent a better habitat, offering greater resource availability and less competition (Baker et al. 2017), while for others (e.g., Chimarra (Curgia)), it may be the only habitat where they develop.

Analyses conducted revealed that Trichoptera assemblages were generally similar nationwide (Fig. 4). Despite the greater number of waterfalls sampled on the Pacific slope, which provided more representation in this study, Northern and Caribbean waterfalls sometimes shared more than 75% similarity in caddisfly diversity with them. Geographic distribution by slope could be a characteristic of some specific caddisflies, as observed among the two subgenera of *Chimarra*. In the group comprising the most significant number of waterfalls, variations among caddisfly assemblages and the associations found between different sites could be more related to changes in the proportion of taxonomic groups, which could also be mediated by factors such as food availability. Suspended organic matter and periphyton constitute the primary food source for macroinvertebrates in waterfalls (Brasil et al. 2014; Baker et al. 2017), so the incidence of sunlight and changes in temperature may favor some groups over others, as well as the magnitude of the flow and the amount of organic matter transported (Clayton and Pearson 2016).

On the other hand, the other assemblages were observed to be largely influenced by site-specific environmental factors. For example, at high altitudes, low temperatures limit the presence of organisms that are not well adapted to these conditions; therefore, diversity may differ from that at low and intermediate altitudes. In the case of site B19, pH could also influence the composition of the assemblage; however, it was the only site with such a low pH value, making it impossible to determine the influence this variable might have on diversity at other altitudes. Furthermore, in intermittent and karst waterfalls, the high solute load transported by both rainfall and rock erosion, reflected in high conductivity values, can generate favorable conditions for some associated organisms. Additionally, intermittent waterfalls may have lower diversity, limited to organisms with faster life cycles that would not be affected by the eventual loss of flow.

One limitation of this study is that caddisfly larvae in our region cannot currently be identified to the species level, as less than 10% of the larvae have been described (Springer, 2010). Analysis on the species level could reveal much clearer differences in the assemblages and highlight even more the uniqueness of waterfalls as a specific habitat. A hint at this is the unique morphotypes found in some larvae, such as those of *Helicopsyche* and *Lepidostoma*. Therefore, future studies on waterfall assemblages should include an effort to associate the larval stages through barcoding and additional adult collecting at each site. Additionally, the associations observed between waterfall Trichoptera assemblages and environmental factors, such as elevation or pH, underscore the importance of including additional sampling sites under the same conditions to better understand these distributional patterns.

Although Trichoptera diversity could not be analyzed at the species level, the preference of some taxa for waterfalls, as shown in this study, highlights the importance of this habitat in maintaining the diversity of rheophilic insects and even hosting unique or rare species (Rackemann et al. 2012). These environments are also important in the face of fluctuations in river flow. With the decrease in flow due to climate change, waterfalls may be the only environment available for rheophilic macroinvertebrates, especially in regions with lower slopes along the basin (Rackemann et al. 2012).

## ﻿Conclusions

This study provides a valuable contribution to our knowledge of Trichoptera diversity associated with waterfalls in a neotropical country. The presence of caddisfly larvae in all studied waterfalls, along with the high diversity reported, indicates that these constitute an important habitat for this diverse aquatic insect group. The high abundance of some genera in the waterfalls, compared to their much lower abundance or absence in the corresponding rivers, indicates a preference for these specific environments, possibly even representing their exclusive habitat. Additionally, some genera or species with similar niches may be adapted to inhabit these two habitats separately, thereby avoiding competition for resources. Waterfall caddisfly diversity was generally similar throughout the country, with differences mediated by site-specific characteristics such as elevation, the geological composition of the bedrock, and chemical factors like temperature and conductivity. The results obtained from this study underscore the importance of expanding research on larval taxonomy and ecology for a deeper understanding of the relationships between species and their habitats of development. This research, which constitutes the first systematic study of macroinvertebrates associated with waterfalls in the Neotropics, highlights the importance of exploring understudied environments to advance our knowledge of freshwater organism ecology and enhance conservation efforts for tropical biodiversity.

## ﻿Additional information

### Use of AI

No use of AI was reported.

### Author ORCIDs

Darha Solano-Ulate https://orcid.org/0009-0002-5581-379X

Monika Springer https://orcid.org/0000-0003-0926-1322

### Data availability

All of the data that support the findings of this study are available in the main text or Supplementary Information.

## Supplementary materials

Supplementary material 1 Number of individuals and relative abundances of caddisfly larvae collected per taxa and sampling site, Wilcoxon test results and additional collecting sites information Data type xlsx https://binary.pensoft.net/file/1488886This dataset is made available under the Open Database License (http://opendatacommons.org/licenses/odbl/1.0/). The Open Database License (ODbL) is a license agreement intended to allow users to freely share, modify, and use this Dataset while maintaining this same freedom for others, provided that the original source and author(s) are credited.Darha Solano-Ulate, Monika Springer
